# How do adjuvants enhance immune responses?

**DOI:** 10.7554/eLife.101259

**Published:** 2024-08-13

**Authors:** Rekha R Rapaka

**Affiliations:** 1 Center for Vaccine Development and Global Health, University of Maryland School of Medicine Baltimore United States; 2 Moderna Therapeutics Cambridge United States

**Keywords:** vaccine, adjuvants, immunization, T cell, Mouse

## Abstract

By altering which peptide antigens are presented to CD4^+^ T cells, adjuvants affect the specificity of the immune response.

**Related research article** Li B, Zhang J, He T, Yuan H, Wu H, Wang P, Wu C. 2024. PRR adjuvants restrain high stability peptides presentation on APCs. *eLife* **13**:RP99173. doi: 10.7554/eLife.99173.

The process by which a vaccine enhances immunity against a disease involves a wide range of cell types. It starts with cells called antigen presenting cells (APCs) internalizing and processing antigens from the vaccine. These APCs then present the antigens on MHC II molecules at the cell surface, a process that can activate cells called T cells. T cell activation is a prerequisite for other immune cells called B cells to produce the antibodies that are crucial to the immune response.

Adjuvants are substances added to vaccines that enhance the immune response to antigens and ultimately, improve immunity. It is known that some, such as MPLA and CpG, directly activate proteins found on APCs called pattern recognition receptors (Reed et al., 2013; Didierlaurent et al., 2014). Yet how adjuvants influence the magnitude and quality of adaptive immune responses through APCs remains unclear. It was recently shown that adding an adjuvant to a vaccine may influence the specific region of a vaccine antigen that an antibody recognizes (Maeda et al., 2017; Chung et al., 2015). This suggests that CD4^+^ T cells, required for fine-tuning B cell antibody responses, are also impacted by adjuvants.

Now, in eLife, Bin Li, Peng Wang, Chao Wu of The Eighth Affiliated Hospital of Sun Yat-sen University and colleagues – including Jin Zhang as joint first author with Li – report the results of experiments which shed light on how adjuvants work (Li et al., 2024). The experiments involved administering a vaccine containing a protein antigen from the bacteria *H. pylori* to mice: in some cases, the vaccine also contained MPLA or CpG as an adjuvant, and in other cases it did not. Interestingly, the various adjuvanted or unadjuvanted vaccination conditions differently impacted which protein sites were most reactive with T cells. When APCs were exposed to *H. pylori* proteins in the presence of these adjuvants, peptide antigens with low affinity for MHC II were more likely to be presented, and the number of different presented peptide sequences was low. In contrast, when the vaccine did not contain adjuvants, peptide antigens with high affinity for MHC II were more likely to be presented, and the number of different presented sequences was higher (Figure 1).

**Figure 1.**
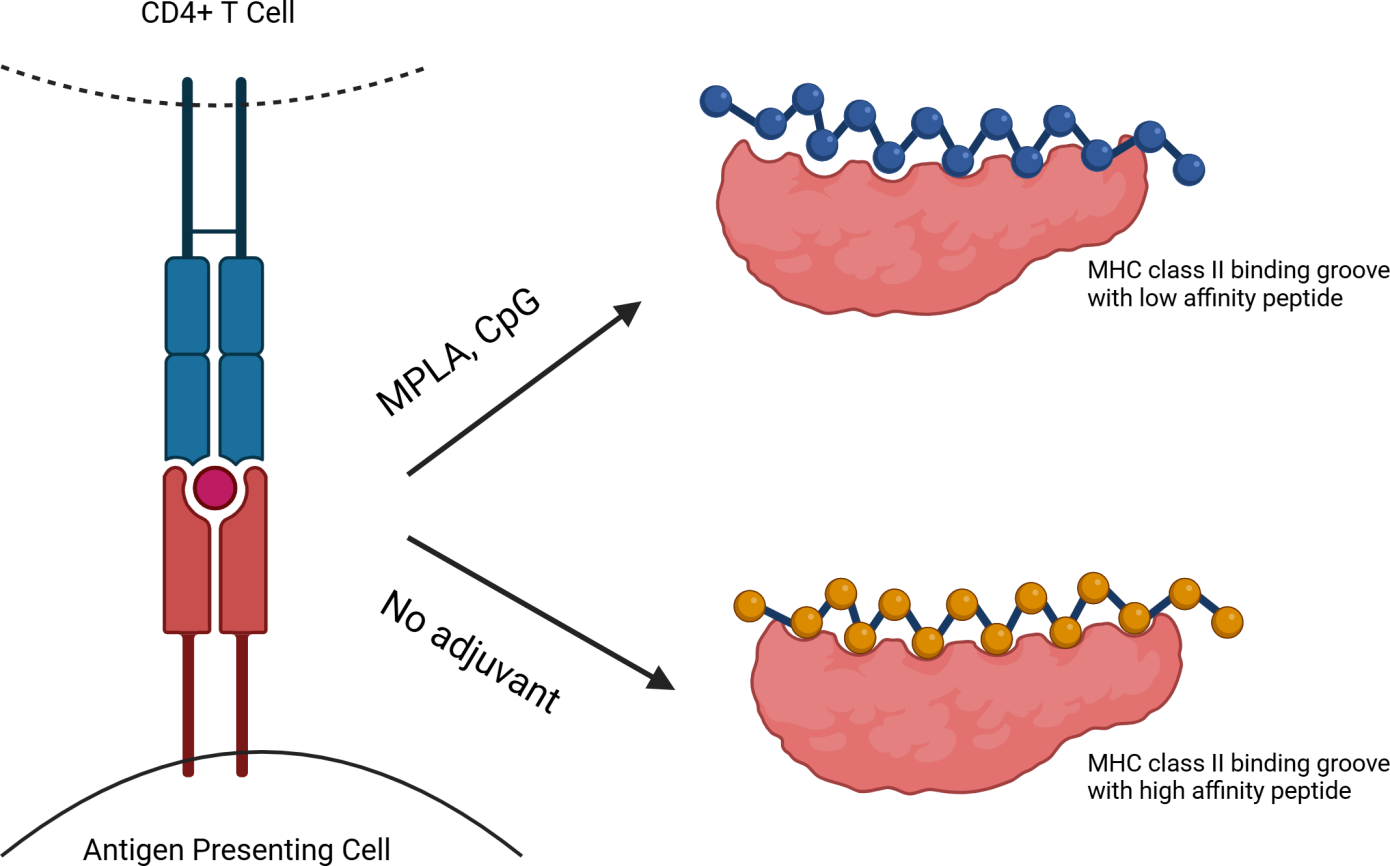
The impact of adjuvants on interactions between APCs and peptide antigens. A CD4^+^ T cell (top left) may be activated when the T cell receptor (blue) interacts with an MHC II molecule (red) and a peptide antigen (red circle) on the surface of an antigen presenting cell (APC; bottom left). APCs exposed to adjuvants (such as MPLA or CpG; top right) in the presence of vaccine antigens present peptide antigens (blue) that have lower affinity molecular interactions with MHC II (pink), and present fewer different peptides. In contrast, in the absence of adjuvants (bottom right), vaccine peptides with higher affinity molecular interactions (yellow) with MHC II are presented, as well as a broader peptide repertoire.

When a peptide with low affinity for MHC II was used to vaccinate mice, it was found that significantly less peptide was needed to activate CD4^+^ T cells compared to responses elicited by vaccination with high-affinity peptides. Taken together, these findings support previous observations that T cell responses to a protein are often focused on a narrow array of peptide antigens (Sant et al., 2005; Baumgartner and Malherbe, 2010), but they also show that different adjuvants have different effects on how T cells respond to peptide antigens. It is an important distinction that adjuvants, beyond increasing the magnitude of a given immune response, may also fine-tune the portion of the antigen targeted by T cells.

How peptide antigens with low affinity for MHC II and a narrow antigen repertoire might impact the CD4^+^ T cell response remains an open question. Some data suggest that the threshold for activating T cells is influenced by adjuvants that can activate pattern recognition receptors, and that the complexes formed by low-affinity peptides and MHC II molecules provide stronger signals to T cell receptors, promoting their activation (Malherbe et al., 2008; Baumgartner et al., 2010).

Alternatively, it has also been shown that a narrow peptide repertoire can enhance T cell responses (Santambrogio, 2022). Could it be that restricting the number of antigens that APCs present (by increasing selection of low-affinity peptides) narrows the peptide repertoire and therefore focuses the T cell response? Li et al. were able to demonstrate that this phenomenon of peptide selection was not due to differences in which antigens were initially taken up by APCs, suggesting that these adjuvants influence antigen processing. Exactly which steps of antigen processing are affected remains to be explored. Might these steps be impacted by pattern recognition receptor signaling? Additionally, it remains to be explored whether these mechanisms are at play in the human immune system, which has more complex MHC II structures than those found in mice.

The findings of Li et al. bring us closer to understanding how adjuvants that activate pattern recognition receptors actually work. By influencing antigen processing and focusing peptide presentation by APCs, adjuvants change the nature of the CD4^+ ^T cell response to a vaccine. This underscores how finely and specifically these adjuvants might influence a vaccine response. Modeling how a given adjuvant fine-tunes the MHC II peptide repertoire may be an important early step in vaccine development. Tackling the development of more challenging vaccines – or optimizing the use of vaccines in populations across the age spectrum – may ultimately rest on our ability to understand and take advantage of the precise mechanisms of these types of adjuvants.
